# The role and mechanism of action of tRNA-derived fragments in the diagnosis and treatment of malignant tumors

**DOI:** 10.1186/s12964-023-01079-3

**Published:** 2023-03-24

**Authors:** Mengdan Gong, Yongqin Deng, Yizhen Xiang, Dong Ye

**Affiliations:** Department of Otorhinolaryngology-Head and Neck Surgery, The Affiliated Lihuili Hospital, Ningbo University, Ningbo, 315040 Zhejiang China

**Keywords:** tRNA-derived fragments, Malignant tumors, Biomarker, Function

## Abstract

Cancer is a leading cause of morbidity and death worldwide. While various factors are established as causing malignant tumors, the mechanisms underlying cancer development remain poorly understood. Early diagnosis and the development of effective treatments for cancer are important research topics. Transfer RNA (tRNA), the most abundant class of RNA molecules in the human transcriptome, participates in both protein synthesis and cellular metabolic processes. tRNA-derived fragments (tRFs) are produced by specific cleavage of pre-tRNA and mature tRNA molecules, which are highly conserved and occur widely in various organisms. tRFs were initially thought to be random products with no physiological function, but have been redefined as novel functional small non-coding RNA molecules that help to regulate RNA stability, modulate translation, and influence target gene expression, as well as other biological processes. There is increasing evidence supporting roles for tRFs in tumorigenesis and cancer development, including the regulation of tumor cell proliferation, invasion, migration, and drug resistance. Understanding the regulatory mechanisms by which tRFs impact these processes has potential to inform malignant tumor diagnosis and treatment. Further, tRFs are expected to become new biological markers for early diagnosis and prognosis prediction in patients with tumors, as well as a targets for precision cancer therapies.

**Video abstract**

**Supplementary Information:**

The online version contains supplementary material available at 10.1186/s12964-023-01079-3.

## Introduction

Cancer is a major public health issue worldwide [1] and an important factor affecting human health and development. The World Health Organization indicated that there were approximately 18.1 million new cancer cases in 2018, resulting in 9.6 million cancer deaths [2]. Cancer diagnosis and treatment were somewhat compromised in 2020, due to the new coronavirus epidemic that began in 2019 [3]. In some developed countries, cancer has become the leading cause of death [4]. Further, as a result of challenges in diagnosing cancer in its early stages, current treatments remain unsatisfactory. Advances in molecular and cell biology technologies have provided a deeper understanding of cancer cell biology, and led to the discovery of additional disease markers. Further, there is growing evidence linking some non-coding RNAs (ncRNAs) to cancer diagnosis and prognosis, highlighting their potential as new predictive tumor markers and therapeutic targets.

ncRNAs occur widely in living organisms, cannot be translated into proteins, and are classified into three categories depending on their length: long (> 200 nucleotides (nt)), medium (> 40 nt), and small (< 40 nt) [5]. Transfer RNAs (tRNAs) are the most abundant class of ncRNA molecules in the human transcriptome, accounting for 4–10% of all RNAs in the cell [6]. The main function of tRNAs is to recognize genetic information and accurately translate it into amino acid sequence information [7]. Recent rapid developments in sequencing technology have led to identification of a new class of small non-coding RNAs (sncRNAs) derived from tRNAs, referred to as tRNA-derived fragments (tRFs), which are specific cleavage products of pre-tRNAs and mature tRNAs and highly conserved evolutionarily [8]. Initially, tRFs were thought to be random products of tRNA turnover, but they have since been shown to occur in various species, be widely distributed in human body fluids [9], and play important roles in several biological processes; hence, exploration of potential tRFs functions is a focus of current research.

In this review, we explore the involvement of tRFs in the structural features and biological functions, as well as mechanisms underlying tumor-related metabolic pathways. We also investigate how tRFs influence metabolism and regulate tumorigenesis and cancer progression. Our report illuminates the potential for application of tRFs as tumor diagnostic biomarkers and in the development of novel therapeutic strategies.

### tRFs structure and biological function

tRFs are the product of tRNA cleavage, and different tRF isoforms are produced depending on where the tRNA is cleaved. These RNA fragments regulate multiple biological processes through a variety of mechanisms, including control of mRNA stability to affect target gene expression and induce translation.

### tRFs structure

In eukaryotes, tRNA genes are transcribed by RNA polymerase III into pre-tRNAs, which bear 5′-leader and 3′-trailer regions [10]. The 5′ and 3′ regions have important features, including the terminal poly-U at the 3′-end of pre-tRNAs, which gives rise to 3′U-tRFs. The steps for conversion of pre-tRNA to mature tRNA molecules involve 5′ and 3′ end processing, intron splicing, common nucleotide modifications, modifications on specific tRNA subsets, aminoacylation, addition of CCA to the processed 3′ end, and nuclear export [11]. Mature tRNAs have a cloverleaf structure of approximately 70–90 nt, consisting of a dihydrouracil loop, a dihydrouracil arm, an anticodon loop, an anticodon arm, a variable loop, a pseudouracil (TψC or T) loop, a pseudouracil arm, and an amino acid arm [12, 13].

Depending on the cleavage site and length, tRNAs produce two isoforms: tRNA-derived stress-induced RNAs (tiRNAs or tRNA halves) and tRNA-derived fragments (tRFs or tDRs) [14]. tiRNAs are usually 28–36 nt in length and are produced by specific ribonucleases (RNases) through endocytosis of the anticodon loop of mature tRNAs [15], which generally occurs under stress conditions [16]. tRFs are around 20 nt in length and are produced by cutting the ends of precursor or mature tRNA molecules [17]. Notably, not all tRNAs are processed into tRFs. Torres et al. found that differential expression of specific tRNA genes can be a mechanism of generating specific tRNA fragments, rather than altering mature tRNA levels [18], suggesting that there may be specific triggers for tRFs production [19].

Since pre-tRNAs and mature tRNAs are cleaved at different locations, the resulting tRFs can be broadly classified into different isoforms [8]: tRF-5, tRF-3, tRF-1, tRF-2, and i-tRF. While tRF-5 and tRF-3 are produced from the 5′ and 3′ ends of mature tRNAs, tRF-1 is produced from the 3′ end of primary tRNAs transcripts [20]. tRF-5 is 14–30 nt in length and is formed by cutting the D-loop, or the stem region between the D- and anticodon loops, of mature tRNA genes. Specific cuts result in three different tRF-5 lengths, that can be further divided into tRF-5 subclasses: tRF-5a (14–16 nt), tRF-5b (22–24 nt), and tRF-5c (28–30 nt). tRF-3 is produced by angiopoietin (ANG), Dicer, or nucleic acid exonuclease digestion of a T-loop, cleaving mature tRNA, and usually contains a CCA tail sequence of approximately 18 or 22 nt, classified as tRF-3a and tRF-3b, respectively [13]. tRF-1 (also referred to as 3′U-tRF) is produced by digestion of the 3′-trailer region of pre-tRNAs by RNase Z or its cytoplasmic homolog, ELAC2 [21], often has a poly-U sequence at its 3′ end, and varies greatly in length [12]. Not all tRNAs can produce all three types of tRFs. While tRF-5 is found primarily in the nucleus, tRF-3 and tRF-1 are mainly localized in the cytoplasm [22]. tRF-2 is generate from by the decomposition of the anticodon loop of tRNA under hypoxic conditions, excluding the 5′ end and the 3′ end structures [7], while i-tRFs originate from within mature tRNAs, although the exact mechanism by which they are produced remains unknown. Small RNAs derived from the 5′-leading and 3′-tailing sequences of pre-tRNAs are also classified as tRFs [17].

Notably, tRNAs cannot be adequately sequenced by standard high-throughput sequencing techniques, due to their abundant post-transcriptional modifications and stable structure, and there is also a lack of accurate, high-resolution computational tools for tRNA quantitation [23, 24]. Fortunately, numerous improved strategies have been developed to address these issues, such as DM-TGIRT-seq (Zheng et al. [23]), Arm-seq (Cozen et al. [25]), Hydro-tRNAseq (Gogakos et al. [26]), AQRNA-seq (Hu et al. [27]), and mim-tRNAseq (Behrens et al. [24]); although these new methods have limitations, they have potential to help solve challenges in understanding tRNA biology.

tRFs can be produced by nucleic acid endonucleases and are closely related to cytoplasmic angiogenin. Under stressful conditions, ANG produces tiRNA by cutting the loops of mature tRNA molecules [28]. The shearing of tRNA by ANG is regulated by 5-methyl-cytosine (m^5^ C)base modification [17, 29, 30]. Further, Alkbh1, an 1-methyl-adenosine demethylase, can also enhance tRNA cleavage and affect tRNA stability [31]. Furthermore, while Dicer is associated with tRF production in some species, others are Dicer-independent [32]. In addition, tRNA introns can serve as recognition elements for the generation of functional tRFs during tRNA splicing [33]. As tRFs are not cleaved into arbitrary sizes or nucleic acid structures, they are likely not randomly generated degradation products [34, 35].

tRNAs undergo a wide range of RNA modifications that affect their structure and related functions [36]; for example, tRNA modifications can determine the specificity of RNase cleavage sites, and have important roles in tRFs/tiRNA biogenesis by regulating tRNA cleavage efficiency to inhibit and promote tRNAs cleavage by specific ribonucleases (RNases) [28, 37].

While tiRNAs are primarily located in the cytoplasm, the location of tRFs is not well defined [38]. Both tRFs and tiRNAs can be released into the extracellular space, either contained in extracellular vesicles or free, by a unknown mechanism, and tRNA and tRF molecules are abundant in extracellular spaces [39–41].

Some researchers have named tRFs derived from specific tRNAs after their parental tRNAs; for example, Wang et al. named a tRF, tRF‑Glu49, as it is spliced from the 49th nucleotide of tRNA‑Glu [42]. Since there are no unified rules for tRF nomenclature, newly identified tRFs have not always been named followed the same convention, causing some confusion in the research community [43]. To address this problem Pliatsika et al. introduced a new labeling scheme, known as the ‘tRF-license plate’, that allows users to associate a tRF with a universal unique label [44]. In this review, we refer to several tRFs using their license plates, where known.

### Biological functions of tRFs

When tRFs were first discovered, they were thought to be random breakdown products with no specific function. tRFs are widely distributed among living creatures, including but not limited to bacteria, viruses, plants [45], and mammals [34], and their highly conserved nature has led some researchers to propose that they evolved earlier than other small RNA molecules [19]. While the functions of most tRFs remain unknown, new studies are beginning to shed some light on their roles in biological processes.

### tRFs regulate mRNA stability to modulate target gene expression

Some tRFs have been shown to be involved in regulating mRNA stability and translation, providing new insights into their roles in disease mechanisms [46]. tRFs have similar properties to microRNAs (miRNAs), a class of sncRNAs that affect mRNA stability by regulating miRNA-induced silencing complex binding to the 3′ untranslated region (UTR) of partially complementary sites in target genes [12]. Some tRFs regulate mRNAs in a miRNA-like manner, by targeting mRNAs, cleaving partially complementary targets, and binding to RNA binding proteins (RBPs), to regulate protein translation by affecting mRNA stability [47]. Huang et al. found that tRF/miR-1280 inhibited colorectal cancer (CRC) cell growth and metastasis by directly interacting with the 3′UTR of its target gene, *JAG2*, and inhibiting Notch signaling [48]. It is important to note that while Dicer is a ribonuclease associated with mature miRNA biogenesis, tRFs is are able to regulate gene expression in a Dicer-dependent or -independent manner [12, 49].

tRFs are closely related to Argonaute (AGO) family proteins, and can regulate gene expression by competitively binding to AGO. tRFs preferentially bind to AGO1, 3, and 4, over AGO 2, in a miRNA-like manner, and may play a major role in RNA silencing, thereby affecting target gene expression [21, 22]. tRFs loading on AGO can be Dicer-dependent or non-dependent [50]. Maute et al. demonstrated that CU1276, a tRNA-derived fragment, can physically associate with AGO proteins and suppress proliferation and modulate the molecular response to DNA damage in B cells in an miRNA-like manner [51]. Further, Kuscu et al. demonstrated that tRF-3, produced upon overexpression of tRNAs, can bind to and regulate mRNAs through RNA-induced silencing complex (RISC)-containing AGO-GW182, thereby suppressing gene expression [52]. Moreover, Guan et al. reported numerous putative interactions between tRFs and introns, and found that tRFs can act as guide molecules for interactions between AGO and agotrons [5].

During respiratory syncytial virus (RSV) infection, RSV leads to tRNA cleavage, resulting in massive induction of tRFs. Deng et al. proposed that the 3′ region of tRF5-GluCTC recognizes a target site in the 3′-untranslated region of *APOER2*, which encodes an anti-RSV protein, suppresses its expression, and consequently promotes RSV replication [53]. Interestingly Choi et al. reported that tRF5-GluCTC depends on AGO proteins, which are central components of RISC in RNA silencing pathways, to regulate genes post-transcriptionally [54]. tRF5-GlyCCC and tRF5-LysCTT can promote RSV replication, and both tRF5-GlyCCC and tRF5-LysCTT function to silence genes in trans. Moreover, the mechanism by which tRF5-GlyCCC represses its target genes differs from that of tRF5-GluCTC, in that tRF5-LysCTT and tRF5-GluCTC use their 3′ regions to recognize the target site, while the 5′ end of tRF5-GlyCCC dominates during gene targeting [55].

### Regulating the translation process

tRNAs are an important class of molecules involved in protein synthesis, and some impact biological activities by regulating translation. tRFs can also regulate protein biosynthesis at the translational level by interfering with assembly of the translation initiation complex; tRFs bind to eIF4G, eIF4A, or the eIF4G/A complex to inhibit translation and induce the assembly of stress granules [43].

tRFs are also involved in ribosome regulation; Gebetsberger et al. demonstrated that they can bind to ribosomes in a stress-dependent manner and inhibit translation in vitro by interfering with peptide bond formation [56]. Further, a Val-tRF from *Haloferax volcanii* competes with mRNA for ribosomes and inhibits translation both in vitro and in vivo, through binding small ribosomal subunits [57, 58]. In the kinetoplastid, *Trypanosoma brucei*, tRF-3A (Thr) is produced in response to nutrient deficiency and binds to ribosomes to regulate translation [45].

Lalande et al. also found that tRFs extracted from *Arabidopsis* leaves and purified inhibited protein synthesis in vitro, suggesting that tRFs may act as translation regulators in plants via a mechanism that is not dependent on sequence complementarity with the target mRNA [45]. In human, Sobala and Hutvagner et al. showed that some 5′ tRFs can inhibit reporter gene translation in vitro and in vivo, with no need for complementary target sites [59]. Another study showed that PUS7-mediated pseudouridylation activates tRFs in stem cells to repress protein translation and regulate stem cell growth and differentiation [60].

### Other functions of tRFs

The presence of tRFs is often associated with cellular stress, immune responses, proliferation, and differentiation [61]. Some tRFs can also regulate cell proliferation and RNA silencing, and specific tRF subpopulations influence the expression of genetic information in mammalian cells [30]. tRFs in the cytoplasm can also enter the mitochondria and potentially participate in mitochondrial biology and related diseases [46].

tRFs have been detected in hematopoietic cells and lymphocytes, where they help to regulate the self-renewal, differentiation, and activation of adult hematopoietic stem and progenitor cells [30], and may function in immune-related activities [17, 29, 62].

In a recent study of glomerular podocytes, Shi et al. showed that tRFs participate in biological processes, such as gene transcription, DNA templating, positive regulation of RNA polymerase II promoter transcription, angiogenesis, and cell adhesion [63]. Further, Su et al. showed that tRFs are associated with tissue specificity, developmental changes, and acute responses to environmental stress [64]. In retrotransposition assays, Schorn et al. found that tRFs strongly repress the two most active long terminal repeat (LTR)-retrotransposons, also known as endogenous retroviruses (ERVs), IAP and MusD/ETn, by targeting the tRNA primer binding site [65].

Avcilar-Kucukgoze et al. proposed that tRF^Arg^ can serve as an arginine donor in protein arginylation and may regulate tRNA^Arg^ partitioning and the balance between protein synthesis and amino acid utilization [66]. Moreover, Torres and Martí proposed that extracellular tRFs are relevant paracrine signaling molecules that can influence gene expression and/or protein translation, to modulate stress and immune responses [39].

In summary, while the biological functions of most tRFs require further investigation, findings to date suggest that they are involved in several regulatory mechanisms in cells, including mRNA stability, translation, and target gene expression. Disease onset and progression are often associated with these mechanisms, suggesting that tRFs could be applied for the development of new therapeutic tools.

### Biological function of tRFs in malignant tumors

tRFs are dynamically regulated during basic cellular activities, and there is growing evidence linking dysregulation of tRFs production to certain human diseases [67]. Aberrant cell proliferation and metastasis are two key features of cancer, and tRFs expression is dysregulated in several malignancies, which may impact cancer cell proliferation, apoptosis, invasion, metastasis, and resistance to treatment [30, 68].

### tRFs regulate malignant tumor invasion and metastasis

As a result of similarities between the two types of sncRNA, some tRFs were mistakenly classified as miRNAs in the early stages of tRF research. MiR-1280, which is thought to inhibit CRC invasion and metastasis by suppressing the target gene, *ROCK1*, is actually derived from tRNA^Leu^, and represents a tRF that inhibits tumor metastasis [29]. Similarly, tRF3008A also inhibits CRC proliferation and migration by interacting with endogenous *FOXK1*, a positive regulator of the Wnt/β-catenin pathway [69]. Moreover, Chen et al. demonstrated that tRF-phe-GAA-031 and tRF-VAL-TCA-002 were significantly associated with distant metastasis of CRC tumor cells [70], while Luan et al. found that tRF-20-M0NK5Y93 promotes CRC cell migration and invasion, partly by regulating Claudin-1 during endothelial cell transformation [71].

**Fig. 1:**
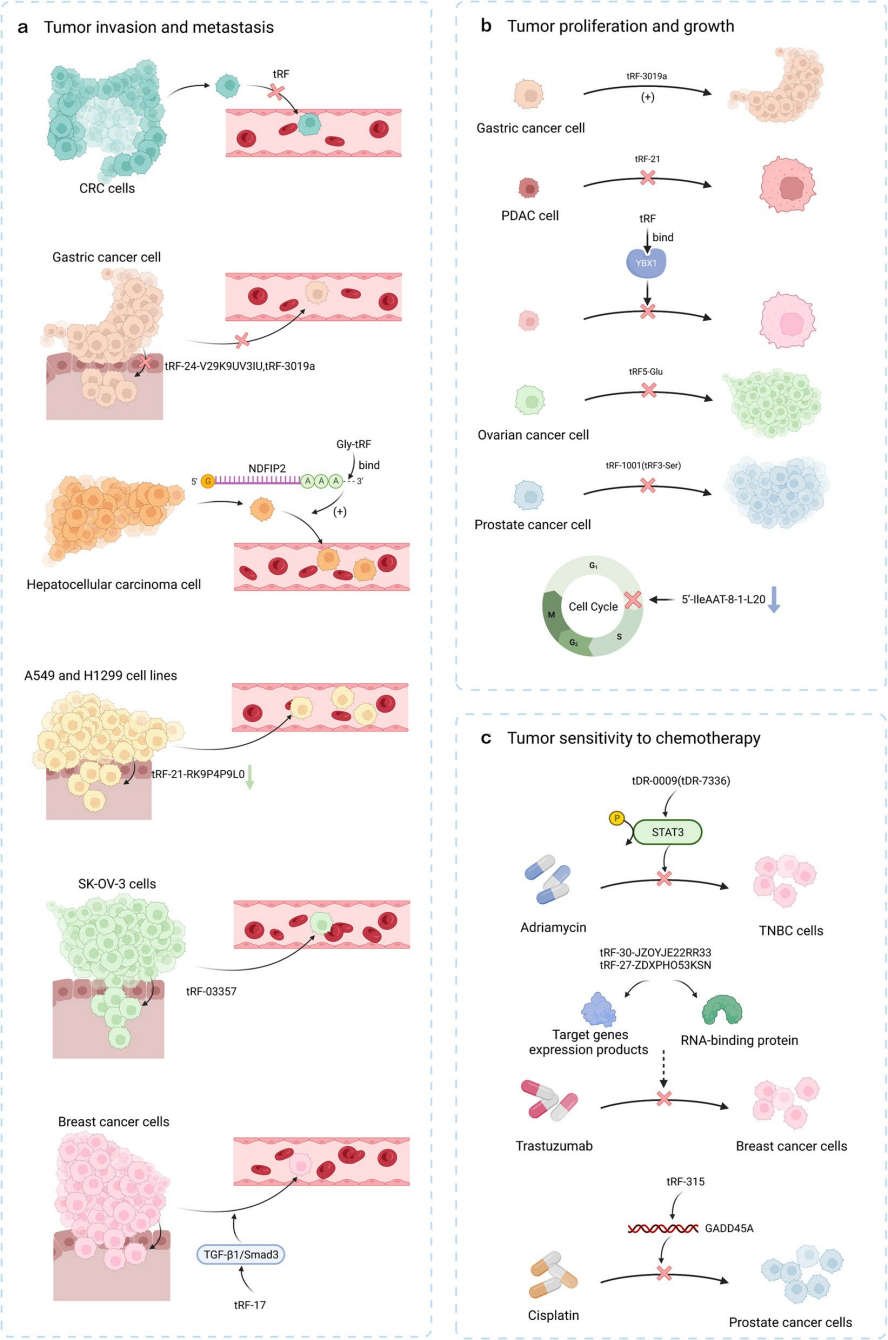
Biological function of tRFs in malignant tumors. **a** tRFs can affect tumor invasion and metastasis in several ways. **b** Overexpression of tRFs in some tumors regulates tumor proliferation and growth, while knockdown shows the opposite consequence. **c** tRFs correlates with radiotherapy sensitivity in some tumors

In gastric cancer (GC) tRF-24-V29K9UV3IU and tRF-3019a inhibit tumor cell migration and invasion and promote apoptosis [68, 72], while in hepatocellular carcinoma, Gly-tRF promotes cell migration and epithelial to mesenchymal transition (EMT) by binding to the 3′UTR of *NDFIP2* mRNA [73]. Further, Wang et al. showed that inhibiting tRF-21-RK9P4P9L0 expression reduced the migration and invasion ability of the A549 and H1299 lung adenocarcinoma cell lines [74]. Zhang et al. found that tRF-03357 promoted the migration and invasion of SK-OV-3 cells, an ovarian cancer (OVCA) cell line [75], while Mo et al. showed that tRF-17 affected breast cancer cell invasion and migration by regulating TGF-β1/Smad3 signaling [76] (Fig. 1a).

### tRFs regulate malignant tumor proliferation and growth

Zhang et al. compared the effects of tRF-3019a knockdown and overexpression on GC cells and found that, while overexpression promoted tumor cell proliferation, knockdown inhibited proliferation, but had no significant effect on the apoptosis rate or cell cycle of GC cells [68].

Pan et al. found that increasing tRF-21 in pancreatic ductal adenocarcinoma cells significantly inhibited cell growth, while decreasing tRF-21 had the opposite effect [77].

There is evidence that tRFs can compete for the binding site of the RBP, YBX1, which has multiple functions in increasing cell proliferation and growth by binding to a collection of endogenous oncogenic transcripts. By binding YBX1, tRFs antagonize YBX1 activity and interfere with oncogenic transcripts, inhibiting tumor growth [78].

Sun et al. found that knockdown of 5′-IleAAT-8-1-L20 suppressed the transition from G1 to S and G2 phases of the cell cycle and validated the oncogenic role of 5′-IleAAT-8-1-L20 in lung cancer [79]. Further, tRF5-Glu mimics inhibited OVCA cell proliferation, while overexpression of tRF-1001 (tRF3-Ser) reduced prostate cancer cell proliferation [80] (Fig. 1b).

### tRFs modulate the sensitivity of malignant tumors to chemotherapy

tRFs can modulate the sensitivity of tumor cells to chemotherapy by regulating related pathways. Cui et al. found that tDRs promote resistance to adriamycin in triple-negative breast cancer (TNBC) and that tDR-0009 (tDR-7336) may contribute to TNBC chemoresistance by inducing STAT3 phosphorylation [81]. Sun et al. found that tRF-30-JZOYJE22RR33 and tRF-27-ZDXPHO53KSN were overexpressed in serum from patients with trastuzumab-resistant breast cancer, and speculated that these two tRFs may be involved in trastuzumab resistance by regulating target gene expression products or binding RBPs [82].

Yang et al. suggested that tRF-315 could target genes such as *GADD45A*, to reduce the sensitivity of prostate cancer cells to cisplatin by inhibiting apoptosis [83]. tRFs can also promote stress granule production, which is associated with cellular drug resistance [84] (Fig. 1c).

In summary, tRFs have regulatory roles in tumor development, and future study of how tRFs can aid tumor diagnostics and treatment is warranted (Fig. 1).

### Diagnostic and therapeutic roles of tRFs in malignant tumors

Most early-stage tumors do not have obvious clinical signs, and the final diagnosis is reliant on an invasive biopsy that may be performed after the optimal window for treatment has passed. Most advanced tumors are treated with surgery, radiotherapy, chemotherapy, and other comprehensive treatments, which can cause patients both physical and mental pain that is sometimes too difficult to withstand. Thus, identifying fast, highly sensitive, and specific early diagnostic markers and efficient and low-risk treatment modalities remain urgently needed by the medical community.

### Diagnostic and prognostic assessment role of tRFs in malignant tumors

While tRFs are defined biological entities, their composition and abundance in the transcriptome vary by gender, race, tissue type, diet, trauma, disease, and disease subtype. Even tRFs length is dependent on tissue type and status [32, 50, 85, 86]. Notably, 3′tRFs expression correlates positively with increasing age, while 5′tRFs expression has no association with age [38]. This suggests that tRFs may serve as potential biomarkers for cancer diagnosis [87].

Xi et al. showed that tRF-1:30-Lys-CTT-1-M2 was significantly upregulated in the extracellular vesicles of patients with hypopharyngeal carcinoma, particularly in those with pulmonary metastases. This has important implications for differentiating between pulmonary from non-pulmonary metastatic hypopharyngeal carcinoma and for differentiating between pulmonary metastases and hypopharyngeal carcinoma [88]. By comparing samples from oral tongue squamous cell carcinoma (SCCHN) patients with those from healthy controls, Gu et al. found that tRF-20-S998LO9D (tRF-20) was significantly upregulated in tumor tissues and that patients with high tRF-20 levels had lower overall survival rates, suggesting that it could serve as a prognostic marker for oral tongue squamous cell carcinoma [89].

Shan et al. found that tRF-39-0VL8K87SIRMM12E2 and tRF-38-0VL8K87SIRMM12V were upregulated in papillary thyroid cancer (PTC), and used repeated qPCR validation to confirm differences in tRF39-0VL8K87SIRMM12E2 expression between thyroid cancer and normal cells. This study revealed that tRF-39-0VL8K-87SIRMM12E2 is primarily involved in metabolic and cancer-related signaling pathways [62].

CRC is the third most common cancer in Western countries. Wu et al. found that CRC patients had significantly higher 5′-tRF-GlyGCC expression than healthy subjects, and both CRC cells and transplanted tumor tissues had higher expression than cells from corresponding controls [9]. Xiong et al. measured 16 tRFs that were differentially expressed in normal and CRC tissues, and differentially expressed(DE) tRFs expression increased during tumor differentiation [90]. Han et al. demonstrated that tRF3008A, a tRF derived from tRNA^Val^, was reduced in CRC and significantly associated with advanced metastasis [69]. Meanwhile, Chen et al. showed that tRF-phe-GAA031 and tRF-VAL-TCA-002 were significantly upregulated in CRC tissues and this correlated with distant tumor metastasis, clinical stage, and survival cycle [70].

Huan et al. found that tRF-31-U5YKFN8DYDZDD was differentially expressed in GC tumor and paraneoplastic tissues, GC patient and healthy human sera, and GC patient sera before and after surgery. It was suggested that tRF-31-U5YKFN8DYDZDD could serve as a GC diagnostic biomarker and a predictor of poor prognosis [91]. Zhang et al. showed that tRF-3019a expression was upregulated in GC tissues and cell lines and could be reliably used to distinguish GC from non-tumor tissues [68]. Gu et al. found that hsa_tsr016141 expression was significantly higher in GC patient tissues and serum and this expression correlated with lymph node metastasis and tumor grade. Hsa_tsr016141 expression was also significantly lower in patient serum after surgery, and the hsa_tsr016141 low expression group had a higher survival rate than the high expression group, indicating that hsa_tsr016141 can effectively predict the postoperative condition of GC patients. Thus, hsa_tsr016141 could be used for early diagnosis of gastric cancer and postoperative monitoring [92]. Xu et al. found that tRF-Glu-TTC-027 was significantly downregulated in GC tissues, and expression correlated with tumor size and histological grade [93]. Shen et al. found that tRF-19-3L7L73JD had lower expression in plasma samples from GC patients than normal subjects, and levels correlated with tumor size [94].

Li et al. found that tRF-Pro-CGG expression was significantly downregulated in pancreatic ductal adenocarcinoma (PDAC) patients, and predicted short clinical survival and poor prognosis. tRF-Pro-CGG may function as a potential biomarker for PDAC progression and treatment [95]. Jin et al. screened four differentially expressed tRFs and tiRNAs from pancreatic cancer and adjacent samples, further validated them by qPCR, and found that while AS-tDR-000064, AS-tDR-000069, and AS-tDR-000102 were upregulated in pancreatic cancer samples, AS-tDR-001391 was downregulated [96].

Wang et al. identified six significantly upregulated tRFs, tRF-Glu-CTC-003, tRF-Gly-CCC-007, tRF-Gly-CCC-008, tRF-Leu-CAA-003, tRF-Ser-TGA-001, and tRF-Ser-TGA-002, in plasma samples from breast cancer patients. HER2 + breast cancer patients with low tRF-Glu-CTC-003 expression had a worse prognosis[97]. Shan et al. found that tRFdb-5024a, 5P_tRNA-Leu-CAA-4-1, and ts-49 expression were positively correlated with the overall survival of breast cancer patients, while ts-34 and ts-58 expression were negatively correlated [98]. Zhang et al. found that tRF-Gly-CCC-046, tRF-Tyr-GTA-010, and tRF-Pro-TGG-001 were downregulated in the tissues and sera of breast cancer patients, and suggested that they may serve as potential biomarkers for early breast cancer diagnosis[99].

Ma et al. found that tRF-Leu-CAG (named as tRF‐LC), a semi-product produced during 5′tRNA process, promoted the development of non-small cell lung cancer (NSCLC), and tRF-LC expression was upregulated during more advanced clinical stages of the disease. Thus, serum tRF-Leu-CAG could be considered for use as a diagnostic biomarker for NSCLC [29]. Wang et al. verified by RT-qPCR that in 37 pairs of lung adenocarcinoma (LUAD) tissues, tRF-16-L85J3KE expression was lower, while tRF-21-RK9P4P9L0 and tRF-16-PSQP4PE expression were higher. tRF-21-RKP4P9L0 was also directly associated with LUAD prognosis [74]. Similarly, Huang et al. used qPCR to assess differences in tRFs expression between lung cancer tissue (LAT) and adjacent normal lung tissue (ANLT) and found that three tRF-1s, tRF-Ser-TGA-010, tRF-Arg-CCT-018, and tRF-Val-CAC-017, had reduced expression in LAT [100]. Gao et al. suggest that 5P_tRNA-SeC-TCA-1-1, 5P_tRNA-Phe-GAA-1-5, 5P_tRNA-Arg-CCG-2-1, and 5c_tRF-Pro-AGG/TGG are correlated with patient prognosis in later stages in lung cancer [101].

Olvedy et al. found that tRFs are differentially expressed in benign prostatic hyperplasia. While tRF-544 expression was lower in high-grade (Gleason score ≥ 7) than low-grade (Gleason score < 7) tumors, tRF-315 was upregulated in high-grade tumors. Thus, the tRF-315/tRF-544 ratio may be a diagnostic marker for disease progression [17]. Moreover, Magee et al. characterize the expression of tRFs in the context of PRAD and suggest tRFs may be related to PRAD biology [102].

Meanwhile, Panoutsopoulou et al. found an association between high expression of internal tRFs derived from tRNA^GlyGCC^ (i-tRF-GlyGCC) and poor prognosis of OVCA patients in addition to short-term disease progression [103]. Papadimitriou et al. found that 5′-tRF-LysCTT (5′-tRF of tRNA^LysCTT^) levels were elevated in bladder cancer (BlCa) and significantly correlated with an aggressive tumor phenotype, early disease progression, and poor treatment outcomes [104]. Londin et al. showed that tRFs fragment length may correlate with patient prognosis. While expression of 18-nt long tRFs was significantly higher in uveal melanoma (UVM) patients with metastasis, expression of 20-nt long tRFs was lower [105]. Xu et al. compared tRFs expression samples from multiple myeloma (MM) patients and healthy donors and confirmed that tRF-60:76-Arg-ACG-1-M2 expression was downregulated in MM patients [106]. After systematically screening 1516 cancer-associated tRFs (ca-tRFs) across seven cancer types Zhao et al. notice that ca-tRFs are not always unidirectional and may be significantly downregulated in cancer samples, but positively correlated with clinical tumor stage [107].

### Therapeutic roles of tRFs in malignant tumors

tRFs may serve as novel biological targets for cancer treatment. Indeed, Kim et al. found that inhibition of a specific tsRNA, LeuCAG3′tsRNA, induces apoptosis in rapidly dividing cells in vitro and in patient-derived mouse models of hepatocellular carcinoma in situ [108], providing a potential new therapeutic cancer target. Shen et al. found that tRF-19-3L7L73JD upregulation in GC cells inhibited their proliferation and migration and promoted apoptosis, suggesting that it may have an inhibitory effect on GC [94]. Dong et al. demonstrated that tRF-24-V29K9UV3IU overexpression also inhibited the proliferation, migration, and invasion of GC cells and promoted their apoptosis [72], while Shen et al. found that tRF-33-P4R8YP9LON4VDP, a representative GC-related tsRNA, may play a tumor-suppressive role in GC and serve as a potential therapeutic target [109].

In prostate cancer, tRF-1001 (tRF3-Ser) overexpression can reduce tumor cell proliferation [80], while Olvedy et al. and Yang et al. found that inhibiting tRF-315 expression led to cellular stress and activation of prostate cancer cell apoptosis [83]. Cao et al. identified a tRF mimetic, tRF-T11 (antisense derived from the 5′ end of tRNA^His(GUG)^ from Chinese yew), that had anticancer activity against OVCA A2780 cells [110]. Moreover, in chronic lymphocytic leukemia samples, 964 tRFs were expressed at levels twofold higher and 701 twofold lower than those in healthy controls, suggesting that dysregulated tRF expression may be associated with the development or progression of this disease [111].

In conclusion, tRFs are aberrantly expressed in tissue or blood samples from patients with cancer and strongly associated with cancer cell proliferation, invasion, and metastasis; thus, tRFs should be considered as a potential prognostic or diagnostic tumor markers as well as therapeutic targets (Table 1). Nevertheless, high-throughput sequencing has inherent limitations as a detection tool, due to sequence-dependent biases in capture, ligation, and amplification during library preparation. Moreover, different data processing and analysis methods can produce varying comparison results, and these aspects require improvement in the future to enhance the relative sensitivity and specificity of tests for tRFs.

**Table 1:** Clinical value of tRFs in cancers

Function	Cancer type	tRFs name/ID	Expression	Effect	References
Potential diagnostic and prognostic predictive utility	Hypopharyngeal carcinoma	tRF-1:30-Lys-CTT-1-M2	Up-regulated		[88]
	SCCHN	tRF-20-S998LO9D	Up-regulated		[89]
	PTC	tRF-39-0VL8K87SIRMM12E2 tRF-38-0VL8K87SIRMM12V	Up-regulated		[62]
	CRC	5’-tRF-GlyGCC	Up-regulated		[9]
		tRF3008A	Down-regulated		[69]
		tRF-phe-GAA031 tRF-VAL-TCA-002	Up-regulated		[70]
	GC	tRF-31-U5YKFN8DYDZDD	Up-regulated		[91]
		tRF-3019a	Up-regulated		[68]
		hsa_tsr016141	Up-regulated		[92]
		tRF-Glu-TTC-027	Down-regulated		[93]
		tRF-19-3L7L73JD	Down-regulated		[94]
	PDAC	tRF-Pro-CGG	Down-regulated		[95]
		AS-tDR-000064 AS-tDR-000069 AS-tDR-000102	Up-regulated		[96]
		AS-tDR-001391	Down-regulated		
	Breast cancer	tRF-Glu-CTC-003 tRF-Gly-CCC-007 tRF-Gly-CCC-008 tRF-Leu-CAA-003 tRF-Ser-TGA-001 tRF-Ser-TGA-002	Up-regulated		[97]
		tRF-Gly-CCC-046 tRF-Tyr-GTA-010 tRF-Pro-TGG-001	Down-regulated		[99]
	Prostate cancer	tRF-544	Down-regulated		[17]
		tRF-315	Up-regulated		
	Ovarian cancer	i-tRF-GlyGCC	up-regulated		[75]
	NSCLC	tRF-Leu-CAG	Up-regulated		[29]
		tRF-16-L85J3KE	Down-regulated		[56]
		tRF-21-RK9P4P9L0 tRF-16-PSQP4PE	Up-regulated		[74]
		tRF-Ser-TGA-010 tRF-Arg-CCT-018 tRF-Val-CAC-017	Down-regulated		[100]
	BlCa	5’-tRF-LysCTT	Up-regulated		[104]
	UVM	18-nt long tRFs	Up-regulated		[105]
		20-nt long tRFs	Down-regulated		
	MM	tRF-60:76-Arg-ACG-1-M2	Down-regulated		[106]
Potential clinical therapeutic targets	Orthotopic hepatocellular carcinoma	LeuCAG3′tsRNA		Apoptosis	[108]
	GC	tRF-19-3L7L73JD		Proliferation,migration, apoptosis	[94]
		tRF-24-V29K9UV3IU		Proliferation, migration invasion, apoptosis	[72]
		tRF-33-P4R8YP9LON4VDP		Antitumor effect	[109]
	Prostate cancer	tRF-1001		Proliferation	[80]
		tRF-315		Apoptosis	[83]
	Ovarian cancer	tRF-T11		Cytotoxicity	[110]

### Regulatory factors and mechanisms of tRFs activity in malignant tumors

The formation and development of malignant tumors is a complex biological process that includes inactivation of oncogenes, overexpression of proto-oncogenes, and disruption of the cell cycle [112]. In addition to understanding the biological functions of tRFs in tumors, clarifying tRF regulatory factors and the related mechanisms by which tumor cell-related biological functions are regulated will be important in determining whether tRFs can become novel diagnostic biomarkers and/or targets for tumor treatment.

### Regulators of tRFs in malignant tumors

When cells are exposed to unfavorable environments, such as hypoxia, oxidative stress, and high salinity, they protect themselves by activating stress response pathways [113], and prolonged exposure to stress can result in disease initiation.

Cui et al. found that tDR-0009 and tDR-7336 expression were significantly upregulated by hypoxic stimulation of TNBC cell lines, possibly related to the chemotherapy resistance mechanism of TNBC that occurs under hypoxic conditions [81]. Such conditions may allow tRFs in breast cancer cells to bind to the oncogenic RBP, YBX1, and inhibit cancer metastasis [77]. Luan et al. found that Dicer1 was overexpressed under hypoxic stimulation and able to promote CRC cell migration and invasion via tRF-20-MEJB5Y13 [49].

By studying yeast, plant cells, and human cell lines, Thompson et al. found that tRNA and rRNA cleavage increases under oxidative stress conditions, and that endonucleolytic cleavage of tRNA molecules may affect tumorigenesis [114]. After exposing the cytosine-5 RNA methyltransferase, NSUN2, to oxidative stress conditions, Gkatza et al. found that NSUN2 activity was inhibited, tRNA methylation reduced, and protein synthesis impaired [115]. Fu et al. demonstrated that various stress conditions, such as nutrient deficiency, hypoxia, and low temperature, induce tRNA cleavage. ANG activates cleaved tRNA and inhibits protein translation during this process [116, 117], thereby regulating malignant tumorigenesis and progression.

### Possible mechanism of tRFs activity in malignant tumors

Roles for tRFs in the progression of human diseases, such as pathological stress injury [118], metabolic disorders [119], immune system disorders [120, 121], viral infections [122], and neurological disorders [123, 124] have been demonstrated; however, it is unclear how tRFs specifically regulate cancer progression. While some tRFs may inhibit cancer progression by regulating oncogene expression, other tRFs promote cell proliferation and cell cycle progression. Indeed, tRFs can regulate the occurrence and progression of breast [29], colorectal [62], gastric [68], liver [73], prostate [83], ovarian [75], and lung [125] cancers.

**Fig. 2:**
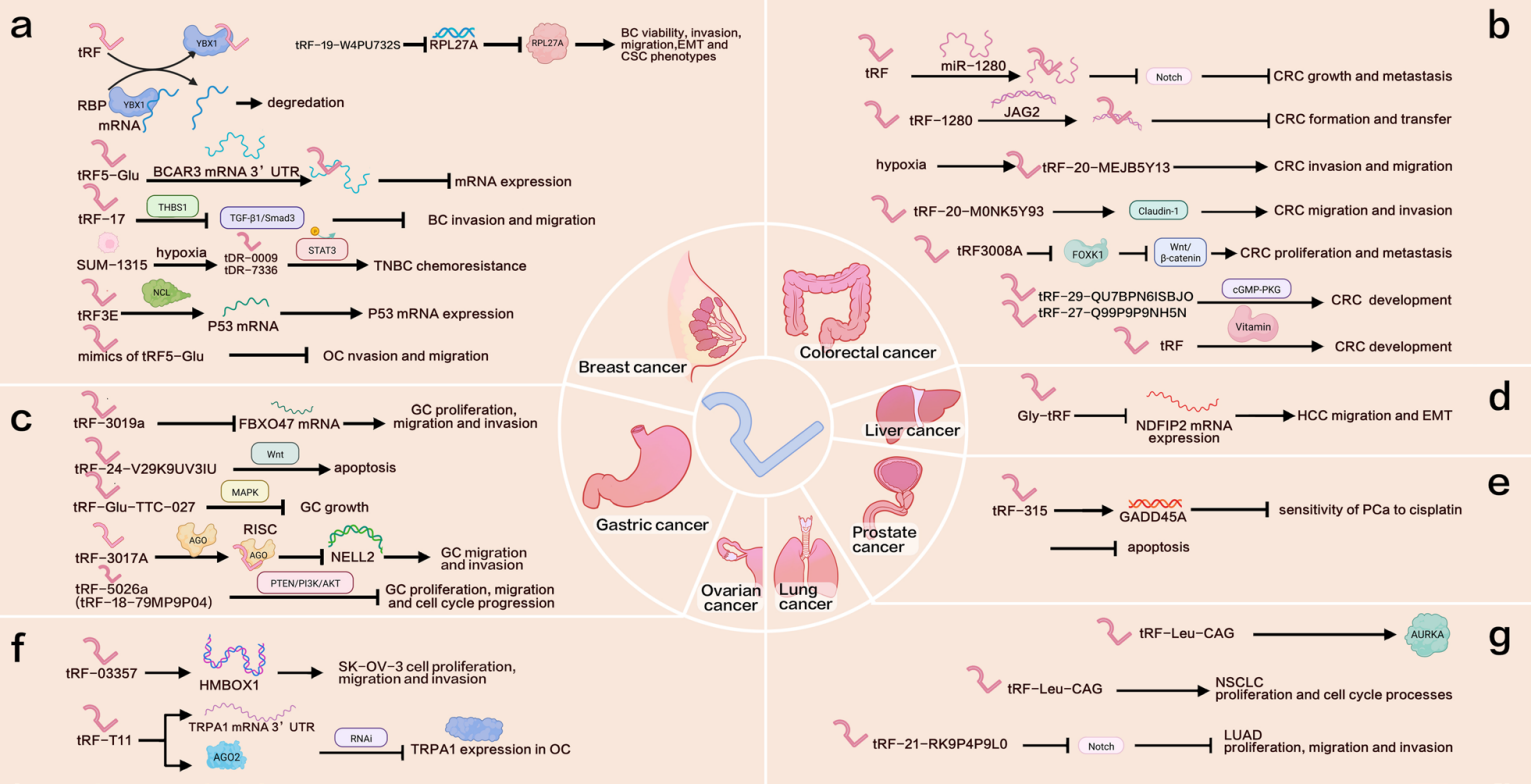
Possible mechanism of action of tRFs in malignant tumors. Various researches have revealed that tRFs have a significant role in tumor progression, but the exact mechanism is not fully understood. tRFs regulate the occurrence and progression of breast, colorectal, gastric, liver, prostate, ovarian, and lung cancers. **a** Mechanism of action of tRFs in breast cancer. **b** Mechanism of action of tRFs in colorectal cancer. **c** Mechanism of action of tRFs in gastric cancer. **d** Mechanism of action of tRFs in liver cancer. **e** Mechanism of action of tRFs in prostate cancer. **f** Mechanism of action of tRFs in ovarian cancer. **g** Mechanism of action of tRFs in lung cancer

In breast cancer research, two mechanisms are thought to impact the development of tRFs: evasion of hypoxia-induced tRFs and upregulation of YBX1 [29]. tRF^Glu^, tRF^Ala^, tRF^Asp^, and tRF^Tyr^ were the first tRFs shown to inhibit breast cancer progression. Goodarzi et al. identified a new class of tRFs produced by tRNA^Glu^, tRNA^Asp^, tRNA^Gly^, and tRNA^Tyr^, which inhibit the stability of multiple oncogenic transcripts in breast cancer cells by binding to and replacing the 3′UTR in the mRNA-binding protein, YBX1, and preventing transcription [78]. YBX1 is highly expressed in many cancers and involved in numerous key cellular pathways. Zhou et al. demonstrated that tRF5-Glu can directly bind to a site in the 3′UTR of breast cancer anti-estrogen resistance gene 3 (*BCAR3*) mRNA and downregulate its expression, while tRF5-Glu mimics can also inhibit OVCA cell proliferation. Thus, tRF5-Glu may provide a new target for therapeutic intervention [80]. Mo et al. found that tRF-17 affects breast cancer cell invasion and migration by attenuating the THBS1-mediated TGF-β1/Smad3 signaling pathway in breast cancer cells, and may serve as a potential target for breast cancer therapy [76]. Cui et al. found that tDR-0009 and tDR-7336, which were significantly upregulated after hypoxic stimulation of SUM-1315 cells, help to maintain stem cell populations and the cellular response to interleukin-6. tDRs promote adriamycin resistance in TNBC, and tDR-0009 (tDR-7336) may be involved in TNBC chemoresistance by activating STAT3 phosphorylation [81]. Falconi et al. demonstrated that tRF3E promotes p53 expression through specific interactions with nucleolin, leading to *p53* mRNA release. In addition, tRF3E is absent from HER2-positive breast cancer, suggesting that it may have a role in the pathogenesis of this disease [126]. Zhang et al. showed that tRF-19-W4PU732S reduced RPL27A expression by directly targeting the 3′UTR, thereby promoting breast cancer cell proliferation, migration, invasion, EMT, and cancer stem cell phenotype capacity [127] (Fig. 2a).

In CRC, a combination of tRFs and miR-1280 inhibits cancer growth and metastasis by suppressing Notch signaling, which supports loss of the stem cell phenotype [62]. By binding to its direct target gene, *JAG2*, tRF-1280 reduces tumor formation and metastasis and suppresses the cancer stem cell phenotype through Notch signaling [29]. Huang et al. found that tRF/miR-1280 reduced CRC growth and metastasis by inhibiting Notch signaling [48], while Luan et al. found that the expression of tRF-20-MEJB5Y13 was increased under hypoxic conditions, and that tRF-20-MEJB5Y13 upregulation by Dicer1 led to hypoxia-induced invasion and migration of CRC cells [49]. Further, Luan et al. found that tRF-20-M0NK5Y93 promotes CRC cell migration and invasion, in part by regulating Claudin-1 during endothelial cell transformation [71]. Furthermore, Xiong et al. suggested that some tRFs, particularly tRF-26-P4R8YP9LOND, tRF-25-P940KK5Y93, tRF-30-XSXMSL73VL4Y, tRF-24-NMEH623K25, and tRF-29-P27JPJ60MVJY, may promote CRC development by regulating vitamin levels. tRF-29-QU7BPN6ISBJO and tRF-27-Q99P9P9NH5N may also be involved in CRC development through cGMP-PKG signaling [90], and Han et al. suggested that tRF3008A affects FOXK1 expression to inhibit Wnt/β-catenin signaling and prevent CRC cell proliferation and metastasis [69] (Fig. 2b).

Studies of GC indicate that tRF-3019a regulates GC cell proliferation, migration, and invasion by targeting FBXO47, and has potential as a diagnostic biomarker for this disease [68], while tRF-24-V29K9UV3IU may promote apoptosis by regulating Wnt signaling [72]. Xu et al. found that tRF-Glu-TTC-027 inhibits GC cell growth through MAPK signaling [93]. Further, Tong et al. found that tRF-3017A regulates the tumor suppressor gene, *NELL2*, by forming a RISC with AGO protein, and inferred that tRF-3017A may promote GC cell migration and invasion by silencing *NELL2* [128]. Moreover, Zhu et al. found that tRF-5026a (tRF-18-79MP9P04) inhibits GC cell proliferation, migration, and cell cycle progression through PTEN/PI3K/AKT signaling, and is a promising diagnostic biomarker for GC [129] (Fig. 2c).

Zhou et al. demonstrated that Gly-tRF can reduce *NDFIP2* mRNA levels by binding to its 3’UTR, thereby promoting hepatocellular carcinoma cell migration and EMT; conversely, Gly-tRF deletion inhibited these processes [73] (Fig. 2d). Yang et al. suggested that tRF-315 reduced the sensitivity of prostate cancer cells to cisplatin by targeting the *GADD45A* gene in prostate cancer cells and regulating apoptosis pathway-related protein expression, to inhibit tumor cell death [83] (Fig. 2e). Zhang et al. showed that tRF-03357 promoted SK-OV-3 highly malignant OVCA cell proliferation, migration, and invasion by regulating HMBOX1 [75]. Using various mechanistic studies, Cao et al. revealed that tRF-T11 can directly target the 3’UTR of *TRPA1* mRNA, or interact with AGO2 to indirectly inhibit TRPA1 expression, in OVCA through the RNAi pathway; TRPA1 is associated with the survival of patients with multiple cancers [110] (Fig. 2f).

Shao et al. showed that tRF-Leu-CAG may be involved in regulating AURKA, while experiments using H1299 cells confirmed that tRF-Leu-CAG could promote non-small cell lung cancer cell proliferation and cell cycle progression [125]. Wang et al. demonstrated that tRF-21-RK9P4P9L0 significantly reduced lung tumor cell proliferation, migration, and invasion by inhibiting Notch 1 expression in a lung adenocarcinoma cell line [74] (Fig. 2g).

Overall, tRFs can regulate tumorigenesis through multiple signaling pathways and impact biological functions related to cell proliferation, invasion, and metastasis (Fig. 2). These features make tRFs promising diagnostic markers and therapeutic targets for cancer.

### Methods for studying tRFs

Many researchers have analyzed tRFs expression data from The Cancer Genome Atlas and developed multiple databases that researchers can access for information [13] (Table 2). Kumar et al. created the first tRFs database, tRFdb, which can be searched by tRF sequence or tRF ID; the advantages of tRFdb are that it can display coordinates and names of retrieved tRFs sequences from various sources [130]. tsRFun was established by Wang et al., and can be used to construct interaction networks among tsRNAs, miRNAs, and mRNAs, offering diverse real-time online tools for tsRNA research [131]. DBtRend provides mature tRNA expression profiles across various biological conditions and enables users to identify biological conditions or tissue types associated with a specific differentially expressed tRNA [132]. tRFexplorer allows users to investigate the potential biological roles of tRNA-derived ncRNAs, without any direct experimental evidence [133]. tRic is the first comprehensive database for tRNAs in cancer, and was developed by Zhang et al.; the database includes codon and amino acid frequency data for all human protein-coding genes [134]. MINTbase v2.0 contains nuclear and mitochondrial tRFs from multiple human tissues [135]. tRF2Cancer can accurately recognize tRFs and evaluate their expression in diverse cancers [136]. Yao et al. developed OncotRF, a tool that can be used to identify diagnostic and prognostic biomarkers for cancers[137]. tsRBase, focuses on tsRNA targets and biological functions, and can help to describe specific tsRNA expression patterns under different backgrounds [138].

**Table 2:** Methods for studying tRFs

Name	Characteristics	Website
tRFdb	Can display coordinates and names of retrieved tRF sequences from various sources	http://genome.bioch.virginia.edu/trfdb
tsRFun	Construct interaction networks among tsRNAs, miRNAs, and mRNAs, offering diverse real-time online tools for tsRNA research	https://rna.sysu.edu.cn/tsRFun/ https://biomed.nscc-gz.cn/DB/tsRFun/
DBtRend	Provides mature tRNA expression profiles across various biological conditions and enables users to identify biological conditions or tissue types associated with a specific differentially expressed tRNA	https://trend.pmrc.re.kr
tRFexplorer	Allows users to investigate the potential biological roles of tRNA-derived ncRNAs, without any direct experimental evidence	https://trfexplorer.cloud/
tRic	Includes codon and amino acid frequency data for all human protein-coding genes	https://hanlab.uth.edu/tRic/ http://bioinfo.life.hust.edu.cn/tRic/
MINTbase v2.0	Contains nuclear and mitochondrial tRFs from multiple human tissues	http://cm.jefferson.edu/MINTbase/
tRF2Cancer	can accurately recognize tRFs and evaluate their expression in diverse cancers	http://rna.sysu.edu.cn/tRFfinder/
OncotRF	can be used to identify diagnostic and prognostic biomarkers for cancers	http://bioinformatics.zju.edu.cn/OncotRF
tsRBase	can help to describe specific tsRNA expression patterns under different backgrounds	http://www.tsrbase.org

## Discussion

As a new type of sncRNAs, tRFs have gradually become the focus of tumor-related research and have received increasing attention in the research community, as their roles in the occurrence and development of tumors and other diseases have gradually been revealed.

Moreover, the function and mechanisms of action of tRFs in diseases have been extensively studied, enriching our understanding of these molecules. As mentioned above, tRFs are aberrantly expressed in various types of human cancer including colon cancer, GC, breast cancer, liver cancer, lung cancer, OVCA, pancreatic ductal adenocarcinoma, prostate cancer, and squamous cell carcinoma of head and neck, and the biological function of tRFs varies among different tumors. For example, in colon cancer, tRFs with tumor-promoting effects include, tRF-20-M0NK5Y93, tRF-20-MEJB5Y13, tRF-26-P4R8YP9LOND, tRF-25-P940KK5Y93, tRF-30-XSXMSL73VL4Y, tRF-24-NMEH623K25, and tRF-29-P27JPJ60MVJY, while tRF-1280 and tRF3008A are among tRFs with inhibitory effects. In GC, tRF-3017A has a tumor-promoting effect, while tRF-24-V29K9UV3IU, tRF-3019a, tRF-19-3L7L73JD, tRF-33-P4R8YP9LON4VDP, tRF-5026a, and tRF-Glu-TTC-027 have inhibitory effects. Similarly, in breast cancer, tDR-7336, tRF-30-JZOYJE22RR33 tRF-27-ZDXPHO53KSN, tDR-0009, tDR-7336, and tRF-19-W4PU732S have tumor-promoting effects, while tRF^Glu^, tRF^Ala^, tRF^Asp^, tRF^Tyr^, tRF5-Glu, and tRF-17 are inhibitory.

Several questions regarding tRFs have yet to be answered. First, numerous tRFs and their mechanisms in tumor development still need to be elucidated and verified through further in-depth studies; for example, the exact relationship between tRFs and miRNAs requires clarification, due to their similar biological characteristics and functions, and ability to regulate target genes. The mechanism by which tRFs can bind protein is also unclear, as is whether corresponding binding sites exist. Second, tRFs nomenclature is currently confusing, and there is an urgent need to develop further unified norms, to provide clarity. Third, as the primary detection tool for tRFs, high-throughput sequencing technologies are known to have strong biases, as well as limitations for the analysis of large clinical samples; hence, tRFs detection methods require further improvement.

## Conclusion

At present, research on tRFs is at a relatively early stage, and deeper understanding of tRFs is needed, while only a small proportion of tRFs have been studied to date. In this review the mechanisms underlying tRFs involvement in various tumors are explored, which both helps to deepen understanding of the functions and properties and provides a new theoretical basis for future exploration of relevant tumor diagnostic markers, disease prognosis assessment, and therapeutic targets. In conclusion, research into tRFs and their function in cancers is likely to be fruitful, and further analyses of tRFs are necessary and will be clinically valuable; hence, tRFs are expected to become a future research focus.

## Data Availability

Not applicable.

## Abbreviations

tRNA: Transfer RNA
tRF: TRNA-derived fragment
sncRNA: Small non-coding RNA
ncRNA: Non-coding RNA
tiRNA: TRNA-derived stress-induced RNA
tsRNA: TRNA-derived small RNA
ANG: Angiogenin
pre-tRNA: Precursor tRNA
m^5^C: 5-Methyl-cytosine
miRNA: MicroRNA
miRISC: MiRNA-induced silencing complex
RBP: RNA binding protein
CRC: Colorectal cancer
AGO: Argonaute
RSV: Respiratory syncytial virus
HSPCs: Adult haematopoietic stem and progenitor cells
ERV: Endogenous retroviruses
GC: Gastric cancer
OVCA: Ovarian cancer
TNBC: Triple-negative breast cancer
SCCHN: Squamous cell carcinoma
PTC: Papillary thyroid cancer
DE: Differentially expressed
PDAC: Pancreatic ductal adenocarcinoma
NSCLC: Non-small cell lung cancer
LUAD: Lung adenocarcinoma
LAT: Lung cancer tissue
ANLT: Adjacent normal lung tissue
BlCa: Bladder cancer
UVM: Uveal melanoma
MM: Multiple myeloma
CLL: Chronic lymphocytic leukemia
BCAR3: Breast cancer anti-estrogen resistance gene 3
IL-6: Interleukin-6
NCL: Nucleolin
CSC: Cancer stem cell
RISC: RNA-induced silencing complex
HGSOC: Highly malignant ovarian cancer

## References

[1] Siegel RL, Miller KD, Jemal A (2019). Cancer statistics, 2019. CA Cancer J Clin.

[2] Bray F, Ferlay J, Soerjomataram I, Siegel RL, Torre LA, Jemal A (2018). Global cancer statistics 2018: GLOBOCAN estimates of incidence and mortality worldwide for 36 cancers in 185 countries. CA Cancer J Clin.

[3] Siegel RL, Miller KD, Fuchs HE, Jemal A (2022). Cancer statistics, 2022. CA Cancer J Clin.

[4] Gyamfi J, Kim J, Choi J (2022). Cancer as a metabolic disorder. Int J Mol Sci.

[5] Guan L, Karaiskos S, Grigoriev A (2020). Inferring targeting modes of argonaute-loaded tRNA fragments. RNA Biol.

[6] Weng Q, Wang Y, Xie Y (2022). Extracellular vesicles-associated tRNA-derived fragments (tRFs): biogenesis, biological functions, and their role as potential biomarkers in human diseases. J Mol Med.

[7] Zhu L, Ge J, Li T, Shen Y, Guo J (2019). tRNA-derived fragments and tRNA halves: the new players in cancers. Cancer Lett.

[8] Sun C, Fu Z, Wang S (2018). Roles of tRNA-derived fragments in human cancers. Cancer Lett.

[9] Wu Y, Yang X, Jiang G (2021). 5′-tRF-GlyGCC: a tRNA-derived small RNA as a novel biomarker for colorectal cancer diagnosis. Genome Med.

[10] Wang Y, Weng Q, Ge J, Zhang X, Guo J, Ye G (2022). tRNA-derived small RNAs: mechanisms and potential roles in cancers. Genes Dis.

[11] Maraia RJ, Lamichhane TN (2011). 3′ processing of eukaryotic precursor tRNAs: 3′ processing of eukaryotic precursor tRNAs. Wiley Interdiscip Rev RNA.

[12] Zong T, Yang Y, Zhao H (2021). tsRNAs: novel small molecules from cell function and regulatory mechanism to therapeutic targets. Cell Prolif.

[13] Xie Y, Yao L, Yu X, Ruan Y, Li Z, Guo J (2020). Action mechanisms and research methods of tRNA-derived small RNAs. SIGNAL Transduct Target Ther.

[14] Balatti V, Nigita G, Veneziano D (2017). tsRNA signatures in cancer. Proc Natl Acad Sci.

[15] Nechooshtan G, Yunusov D, Chang K, Gingeras TR (2020). Processing by RNase 1 forms tRNA halves and distinct Y RNA fragments in the extracellular environment. Nucleic Acids Res.

[16] Pablo Tosar J, Segovia M, Castellano M (2020). Fragmentation of extracellular ribosomes and tRNAs shapes the extracellular RNAome. Nucleic Acids Res.

[17] Olvedy M, Scaravilli M, Hoogstrate Y, Visakorpi T, Jenster G, Martens ES (2016). A comprehensive repertoire of tRNA-derived fragments in prostate cancer. Oncotarget.

[18] Torres AG, Reina O, Stephan-Otto Attolini C, Ribas de Pouplana L (2019). Differential expression of human tRNA genes drives the abundance of tRNA-derived fragments. Proc Natl Acad Sci USA.

[19] Krishna S, Raghavan S, DasGupta R, Palakodeti D (2021). tRNA-derived fragments (tRFs): establishing their turf in post-transcriptional gene regulation. Cell Mol Life Sci.

[20] Kumar P, Kuscu C, Dutta A (2016). Biogenesis and function of transfer RNA related fragments (tRFs). Trends Biochem Sci.

[21] Li S, Xu Z, Sheng J (2018). tRNA-derived small RNA: a novel regulatory small non-coding RNA. Genes.

[22] Kumar P, Anaya J, Mudunuri SB, Dutta A (2014). Meta-analysis of tRNA derived RNA fragments reveals that they are evolutionarily conserved and associate with AGO proteins to recognize specific RNA targets. BMC Biol.

[23] Zheng G, Qin Y, Clark WC, et al. Efficient and quantitative high-throughput tRNA sequencing. Nat Methods. 2015;12(9):835-+. 10.1038/NMETH.3478. 10.1038/nmeth.3478 PMC4624326 26214130

[24] Behrens A, Rodschinka G, Nedialkova DD (2021). High-resolution quantitative profiling of tRNA abundance and modification status in eukaryotes by mim-tRNAseq. Mol Cell.

[25] Cozen AE, Quartley E, Holmes AD, Hrabeta-Robinson E, Phizicky EM, Lowe TM. ARM-seq: AlkB-facilitated RNA methylation sequencing reveals a complex landscape of modified tRNA fragments. Nat Methods. 2015;12(9):879-+. 10.1038/NMETH.3508. 10.1038/nmeth.3508 PMC4553111 26237225

[26] Gogakos T, Brown M, Garzia A, Meyer C, Hafner M, Tuschl T (2017). Characterizing expression and processing of precursor and mature human tRNAs by hydro-tRNAseq and PAR-CLIP. Cell Rep.

[27] Hu JF, Yim D, Ma D, et al. Quantitative mapping of the cellular small RNA landscape with AQRNA-seq. Nat Biotechnol. 2021;39(8):978-+. 10.1038/s41587-021-00874-y 10.1038/s41587-021-00874-y PMC8355021 33859402

[28] Akiyama Y, Kharel P, Abe T, Anderson P, Ivanov P. Isolation and initial structure-functional characterization of endogenous tRNA-derived stress-induced RNAs. RNA Biol. 17(8):1116–1124. 10.1080/15476286.2020.1732702 10.1080/15476286.2020.1732702 PMC7549717 32116132

[29] Ma Z, Zhou J, Shao Y, Jafari FA, Qi P, Li Y (2020). Biochemical properties and progress in cancers of tRNA-derived fragments. J Cell Biochem.

[30] Guzzi N, Bellodi C (2020). Novel insights into the emerging roles of tRNA-derived fragments in mammalian development. RNA Biol.

[31] Rashad S, Han X, Sato K (2020). The stress specific impact of ALKBH1 on tRNA cleavage and tiRNA generation. RNA Biol.

[32] Magee R, Londin E, Rigoutsos I (2019). TRNA-derived fragments as sex-dependent circulating candidate biomarkers for Parkinson’s disease. Parkinsonism Relat Disord.

[33] Hanada T, Weitzer S, Mair B (2013). CLP1 links tRNA metabolism to progressive motor-neuron loss. Nature.

[34] Shen Y, Yu X, Zhu L, Li T, Yan Z, Guo J (2018). Transfer RNA-derived fragments and tRNA halves: biogenesis, biological functions and their roles in diseases. J Mol Med.

[35] Kim HK (2019). Transfer RNA-derived small non-coding RNA: dual regulator of protein synthesis. Mol Cells.

[36] Li J, Shen Z, Luo L (2021). tRNA Ini CAT inhibits proliferation and promotes apoptosis of laryngeal squamous cell carcinoma cells. J Clin Lab Anal.

[37] Lyons SM, Fay MM, Ivanov P (2018). The role of RNA modifications in the regulation of tRNA cleavage. FEBS Lett.

[38] Jia Y, Tan W, Zhou Y (2020). Transfer RNA-derived small RNAs: potential applications as novel biomarkers for disease diagnosis and prognosis. Ann Transl Med.

[39] Torres AG, Martí E (2021). Toward an understanding of extracellular tRNA biology. Front Mol Biosci.

[40] Zhu L, Li J, Gong Y (2019). Exosomal tRNA-derived small RNA as a promising biomarker for cancer diagnosis. Mol Cancer.

[41] Tosar JP, Cayota A (2020). Extracellular tRNAs and tRNA-derived fragments. RNA Biol.

[42] Wang Y, Xia W, Shen F, Zhou J, Gu Y, Chen Y (2022). tRNA-derived fragment tRF-Glu49 inhibits cell proliferation, migration and invasion in cervical cancer by targeting FGL1. Oncol Lett.

[43] Li S, Xu Z, Sheng J (2018). tRNA-derived small RNA: a novel regulatory small non-coding RNA. Genes.

[44] Pliatsika V, Loher P, Telonis AG, Rigoutsos I (2016). MINTbase: a framework for the interactive exploration of mitochondrial and nuclear tRNA fragments. Bioinformatics.

[45] Lalande S, Merret R, Salinas-Giege T, Drouard L (2020). Arabidopsis tRNA-derived fragments as potential modulators of translation. RNA Biol.

[46] Cao J, Cowan DB, Wang DZ (2020). tRNA-derived small RNAs and their potential roles in cardiac hypertrophy. Front Pharmacol.

[47] Yu X, Xie Y, Zhang S, Song X, Xiao B, Yan Z (2021). tRNA-derived fragments: mechanisms underlying their regulation of gene expression and potential applications as therapeutic targets in cancers and virus infections. Theranostics.

[48] Huang B, Yang H, Cheng X (2017). tRF/miR-1280 suppresses stem cell–like cells and metastasis in colorectal cancer. Cancer Res.

[49] Luan N, Mu Y, Mu J (2021). Dicer1 Promotes Colon Cancer Cell Invasion and Migration Through Modulation of tRF-20-MEJB5Y13 Expression Under Hypoxia. Front Genet.

[50] Telonis AG, Loher P, Magee R (2019). tRNA fragments show intertwining with mRNAs of specific repeat content and have links to disparities. Cancer Res.

[51] Maute RL, Schneider C, Sumazin P (2013). tRNA-derived microRNA modulates proliferation and the DNA damage response and is down-regulated in B cell lymphoma. Proc Natl Acad Sci.

[52] Kuscu C, Kumar P, Kiran M, Su Z, Malik A, Dutta A (2018). tRNA fragments (tRFs) guide Ago to regulate gene expression post-transcriptionally in a Dicer-independent manner. RNA.

[53] Deng J, Ptashkin RN, Chen Y (2015). Respiratory syncytial virus utilizes a tRNA fragment to suppress antiviral responses through a novel targeting mechanism. Mol Ther.

[54] Choi EJ, Ren J, Zhang K (2020). The importance of AGO 1 and 4 in post-transcriptional gene regulatory function of tRF5-GluCTC, an respiratory syncytial virus-induced tRNA-derived RNA fragment. Int J Mol Sci.

[55] Zhou L, Liu S, Chen Y (2017). Identification of two novel functional tRNA-derived fragments induced in response to respiratory syncytial virus infection. J Gen Virol.

[56] Gebetsberger J, Zywicki M, Künzi A, Polacek N (2012). tRNA-derived fragments target the ribosome and function as regulatory non-coding RNA in haloferax volcanii. Archaea.

[57] Park J, Ahn SH, Shin MG, Kim HK, Chang S (2020). tRNA-derived small RNAs: novel epigenetic regulators. Cancers.

[58] Gebetsberger J, Wyss L, Mleczko AM, Reuther J, Polacek N (2017). A tRNA-derived fragment competes with mRNA for ribosome binding and regulates translation during stress. RNA Biol.

[59] Sobala A, Hutvagner G (2013). Small RNAs derived from the 5 end of tRNA can inhibit protein translation in human cells. Rna Biol.

[60] Guzzi N, Cieśla M, Ngoc PCT (2018). Pseudouridylation of tRNA-derived fragments steers translational control in stem cells. Cell.

[61] Drino A, Oberbauer V, Troger C (2020). Production and purification of endogenously modified tRNA-derived small RNAs. RNA Biol.

[62] Shan S, Wang Y, Zhu C (2020). A comprehensive expression profile of tRNA-derived fragments in papillary thyroid cancer. J Clin Lab Anal.

[63] Shi H, Yu M, Wu Y (2020). tRNA-derived fragments (tRFs) contribute to podocyte differentiation. Biochem Biophys Res Commun.

[64] Su Z, Frost EL, Lammert CR, Przanowska RK, Lukens JR, Dutta A (2020). tRNA-derived fragments and microRNAs in the maternal-fetal interface of a mouse maternal-immune-activation autism model. RNA Biol..

[65] Schorn AJ, Gutbrod MJ, LeBlanc C, Martienssen R (2017). LTR-Retrotransposon Control by tRNA-Derived Small RNAs. Cell.

[66] Avcilar-Kucukgoze AJ, Gamper MJ, Polte C (2020). tRNAArg-derived fragments can serve as arginine donors for protein arginylation. Cell Chem Biol..

[67] Gonskikh Y, Gerstl M, Kos M (2020). Modulation of mammalian translation by a ribosome-associated tRNA half. RNA Biol.

[68] Zhang F, Shi J, Wu Z (2020). A 3′-tRNA-derived fragment enhances cell proliferation, migration and invasion in gastric cancer by targeting FBXO47. Arch Biochem Biophys.

[69] Han Y, Peng Y, Liu S (2022). tRF3008A suppresses the progression and metastasis of colorectal cancer by destabilizing FOXK1 in an AGO-dependent manner. J Exp Clin Cancer Res.

[70] Chen H, Xu Z, Cai H, Peng Y, Yang L, Wang Z (2022). Identifying differentially expressed tRNA-derived small fragments as a biomarker for the progression and metastasis of colorectal cancer. Dis Mark.

[71] Luan N, Chen Y, Li Q (2021). TRF-20-M0NK5Y93 suppresses the metastasis of colon cancer cells by impairing the epithelial-to-mesenchymal transition through targeting Claudin-1. Am J Transl Res.

[72] Dong X, Fan X, He X (2020). Comprehensively identifying the key tRNA-derived fragments and investigating their function in gastric cancer processes. OncoTargets Ther.

[73] Zhou Y, Hu J, Liu L (2021). Gly-tRF enhances LCSC-like properties and promotes HCC cells migration by targeting NDFIP2. Cancer Cell Int.

[74] Wang J, Liu X, Cui W (2022). Plasma tRNA-derived small RNAs signature as a predictive and prognostic biomarker in lung adenocarcinoma. Cancer Cell Int.

[75] Zhang M, Li F, Wang J (2019). tRNA-derived fragment tRF-03357 promotes cell proliferation, migration and invasion in high-grade serous ovarian cancer. OncoTargets Ther.

[76] Mo D, He F, Zheng J, Chen H, Tang L, Yan F (2021). tRNA-derived fragment tRF-17-79MP9PP attenuates cell invasion and migration via THBS1/TGF-β1/Smad3 axis in breast cancer. Front Oncol.

[77] Pan L, Huang X, Liu ZX (2021). Inflammatory cytokine-regulated tRNA-derived fragment tRF-21 suppresses pancreatic ductal adenocarcinoma progression. J Clin Invest.

[78] Goodarzi H, Liu X, Nguyen HCB, Zhang S, Fish L, Tavazoie SF (2015). Endogenous tRNA-derived fragments suppress breast cancer progression via YBX1 displacement. Cell.

[79] Sun X, Yang J, Yu M (2020). Global identification and characterization of tRNA-derived RNA fragment landscapes across human cancers. NAR Cancer.

[80] Zhou K, Diebel KW, Holy J (2017). A tRNA fragment, tRF5-Glu, regulates BCAR3 expression and proliferation in ovarian cancer cells. Oncotarget.

[81] Cui Y, Huang Y, Wu X (2019). Hypoxia-induced tRNA-derived fragments, novel regulatory factor for doxorubicin resistance in triple-negative breast cancer. J Cell Physiol.

[82] Sun C, Yang F, Zhang Y (2018). tRNA-derived fragments as novel predictive biomarkers for trastuzumab-resistant breast cancer. Cell Physiol Biochem Int J Exp Cell Physiol Biochem Pharmacol.

[83] Yang C, Lee M, Song G, Lim W (2021). tRNALys-derived fragment alleviates cisplatin-induced apoptosis in prostate cancer cells. Pharmaceutics.

[84] Zhang Y, Qian H, He J, Gao W (2020). Mechanisms of tRNA-derived fragments and tRNA halves in cancer treatment resistance. Biomark Res.

[85] Telonis AG, Loher P, Honda S (2015). Dissecting tRNA-derived fragment complexities using personalized transcriptomes reveals novel fragment classes and unexpected dependencies. Oncotarget.

[86] Telonis AG, Rigoutsos I (2018). Race disparities in the contribution of miRNA isoforms and tRNA-derived fragments to triple-negative breast cancer. Cancer Res.

[87] Goodarzi H, Nguyen HCB, Zhang S, Dill BD, Molina H, Tavazoie SF (2016). Modulated expression of specific tRNAs drives gene expression and cancer progression. Cell.

[88] Xi J, Zeng Z, Li X, Zhang X, Xu J (2021). Expression and diagnostic value of tRNA-derived fragments secreted by extracellular vesicles in hypopharyngeal carcinoma. Oncotargets Ther.

[89] Gu X, Wang L, Coates P, et al. Transfer-RNA-derived fragments are potential prognostic factors in patients with squamous cell carcinoma of the head and neck. Genes. 2020;11(11). 10.3390/genes11111344 10.3390/genes11111344 PMC7698123 33202812

[90] Xiong W, Wang X, Cai X (2019). Identification of tRNA-derived fragments in colon cancer by comprehensive small RNA sequencing. Oncol Rep.

[91] Huang Y, Zhang H, Gu X (2021). Elucidating the role of serum tRF-31-U5YKFN8DYDZDD as a novel diagnostic biomarker in gastric cancer (GC). Front Oncol.

[92] Gu X, Ma S, Liang B, Ju S (2021). Serum hsa_tsr016141 as a Kind of tRNA-derived fragments is a novel biomarker in gastric cancer. Front Oncol.

[93] Xu W, Zhou B, Wang J (2021). tRNA-derived fragment tRF-Glu-TTC-027 regulates the progression of gastric carcinoma via MAPK signaling pathway. Front Oncol.

[94] Shen Y, Xie Y, Yu X (2021). Clinical diagnostic values of transfer RNA-derived fragment tRF-19-3L7L73JD and its effects on the growth of gastric cancer cells. J Cancer.

[95] Li J, Jin L, Gao Y (2021). Low expression of tRF-Pro-CGG predicts poor prognosis in pancreatic ductal adenocarcinoma. J Clin Lab Anal.

[96] Jin L, Zhu C, Qin X (2019). Expression profile of tRNA-derived fragments in pancreatic cancer. Oncol Lett.

[97] Wang J, Ma G, Li M (2020). Plasma tRNA fragments derived from 5′ ends as novel diagnostic biomarkers for early-stage breast cancer. Mol Ther Nucleic Acids.

[98] Shan N, Li N, Dai Q, et al. Interplay of tRNA-derived fragments and T cell activation in breast cancer patient survival. Cancers. 2020;12(8). 10.3390/cancers12082230. 10.3390/cancers12082230 PMC7466003 32785169

[99] Zhang Y, Bi Z, Dong X (2021). tRNA-derived fragments: tRF-Gly-CCC-046, tRF-Tyr-GTA-010 and tRF-Pro-TGG-001 as novel diagnostic biomarkers for breast cancer. Thorac Cancer.

[100] Huang LT, Cui M, Silva M (2022). Expression profiles of tRNA-derived fragments and their potential roles in lung adenocarcinoma. Ann Transl Med..

[101] Gao Z, Jijiwa M, Nasu M (2022). Comprehensive landscape of tRNA-derived fragments in lung cancer. Mol Ther Oncolytics.

[102] Magee RG, Telonis AG, Loher P, Londin E, Rigoutsos I (2018). Profiles of miRNA isoforms and tRNA fragments in prostate cancer. Sci Rep.

[103] Panoutsopoulou K, Dreyer T, Dorn J (2022). tRNAGlyGCC-derived internal fragment (i-tRF-GlyGCC) in ovarian cancer treatment outcome and progression. Cancers.

[104] Papadimitriou MA, Avgeris M, Levis P (2020). tRNA-derived fragments (tRFs) in bladder cancer: increased 5′-tRF-LysCTT results in disease early progression and patients’ poor treatment outcome. Cancers.

[105] Londin E, Magee R, Shields CL, Lally SE, Sato T, Rigoutsos I (2020). IsomiRs and tRNA-derived fragments are associated with metastasis and patient survival in uveal melanoma. Pigment Cell Melanoma Res.

[106] Xu C, Fu Y (2021). Expression profiles of tRNA-derived fragments and their potential roles in multiple myeloma. OncoTargets Ther.

[107] Zhou Y, Cui Q, Zhou Y (2022). Screening and comprehensive analysis of cancer-associated tRNA-derived fragments. Front Genet.

[108] Kim HK, Fuchs G, Wang S (2017). A transfer-RNA-derived small RNA regulates ribosome biogenesis. Nature.

[109] Shen Y, Yu X, Ruan Y (2021). Global profile of tRNA-derived small RNAs in gastric cancer patient plasma and identification of tRF-33-P4R8YP9LON4VDP as a new tumor suppressor. Int J Med Sci.

[110] Cao KY, Yan TM, Zhang JZ (2022). A tRNA-derived fragment from Chinese yew suppresses ovarian cancer growth via targeting TRPA1. Mol Ther Nucleic Acids.

[111] Veneziano D, Tomasello L, Balatti V (2019). Dysregulation of different classes of tRNA fragments in chronic lymphocytic leukemia. Proc Natl Acad Sci USA.

[112] Ye D, Gong M, Deng Y (2022). Roles and clinical application of exosomal circRNAs in the diagnosis and treatment of malignant tumors. J Transl Med.

[113] Lyons SM, Kharel p, Akiyama y (2020). eIF4G has intrinsic G-quadruplex binding activity that is required for tiRNA function. Nucleic Acids Res.

[114] Thompson DM, Lu C, Green PJ, Parker R (2008). tRNA cleavage is a conserved response to oxidative stress in eukaryotes. RNA.

[115] Gkatza NA, Castro C, Harvey RF (2019). Cytosine-5 RNA methylation links protein synthesis to cell metabolism. PLOS Biol.

[116] Fu H, Feng J, Liu Q (2009). Stress induces tRNA cleavage by angiogenin in mammalian cells. FEBS Lett.

[117] Yamasaki S, Ivanov P, Hu GF, Anderson P (2009). Angiogenin cleaves tRNA and promotes stress-induced translational repression. J Cell Biol.

[118] Qin C, Feng H, Zhang C, et al. Differential Expression Profiles and Functional Prediction of tRNA-Derived Small RNAs in Rats After Traumatic Spinal Cord Injury. Front Mol Neurosci. 2020;12. 10.3389/fnmol.2019.00326 10.3389/fnmol.2019.00326 PMC6968126 31998075

[119] Chen Q, Yan M, Cao Z (2016). Sperm tsRNAs contribute to intergenerational inheritance of an acquired metabolic disorder. Science.

[120] Xu H, Chen W, Zheng F (2020). The potential role of tRNAs and small RNAs derived from tRNAs in the occurrence and development of systemic lupus erythematosus. Biochem Biophys Res Commun.

[121] Zhang Y, Cai F, Liu J (2018). Transfer RNA-derived fragments as potential exosome tRNA-derived fragment biomarkers for osteoporosis. Int J Rheum Dis.

[122] Wang Q, Lee I, Ren J, Ajay SS, Lee YS, Bao X (2013). Identification and functional characterization of tRNA-derived RNA fragments (tRFs) in respiratory syncytial virus infection. Mol Ther.

[123] Qin C, Xu PP, Zhang X (2020). Pathological significance of tRNA-derived small RNAs in neurological disorders. Neural Regen Res.

[124] Puhakka N, Das Gupta S, Vuokila N, Pitkänen A (2022). Transfer RNA-derived fragments and isomiRs are novel components of chronic TBI-induced neuropathology. Biomedicines.

[125] Shao Y, Sun Q, Liu X, Wang P, Wu R, Ma Z (2017). tRF-Leu-CAG promotes cell proliferation and cell cycle in non-small cell lung cancer. Chem Biol Drug Des.

[126] Falconi M, Giangrossi M, Zabaleta ME (2019). A novel 3′-tRNA(GIu)-derived fragment acts as a tumor suppressor in breast cancer by targeting nucleolin. FASEB J.

[127] Zhang Z, Liu Z, Zhao W, Zhao X, Tao Y (2022). tRF-19-W4PU732S promotes breast cancer cell malignant activity by targeting inhibition of RPL27A (ribosomal protein-L27A). Bioengineered.

[128] Tong L, Zhang W, Qu B (2021). The tRNA-derived fragment-3017A promotes metastasis by inhibiting NELL2 in human gastric cancer. Front Oncol.

[129] Zhu L, Li Z, Yu X (2021). The tRNA-derived fragment 5026a inhibits the proliferation of gastric cancer cells by regulating the PTEN/PI3K/AKT signaling pathway. Stem Cell Res Ther.

[130] Kumar P, Mudunuri S, Anaya J, Dutta A (2015). tRFdb: a database for transfer RNA fragments. Nucleic Acids Res.

[131] Wang JH, Chen WX, Mei SQ (2022). tsRFun: a comprehensive platform for decoding human tsRNA expression, functions and prognostic value by high-throughput small RNA-Seq and CLIP-Seq data. Nucleic Acids Res.

[132] Lee JO, Lee M, Chung YJ (2021). DBtRend: a web-server of tRNA expression profiles from small RNA sequencing data in humans. Genes.

[133] La Ferlita A, Alaimo S, Veneziano D (2019). Identification of tRNA-derived ncRNAs in TCGA and NCI-60 panel cell lines and development of the public database tRFexplorer. Database J Biol Databases Curation.

[134] Zhang Z, Ruan H, Liu CJ (2020). tRic: a user-friendly data portal to explore the expression landscape of tRNAs in human cancers. RNA Biol.

[135] Pliatsika V, Loher P, Magee R (2018). MINTbase v2.0: a comprehensive database for tRNA-derived fragments that includes nuclear and mitochondrial fragments from all The Cancer Genome Atlas projects. Nucleic Acids Res.

[136] Zheng LL, Xu WL, Liu S (2016). tRF2Cancer: A web server to detect tRNA-derived small RNA fragments (tRFs) and their expression in multiple cancers. Nucleic Acids Res.

[137] Yao D, Sun X, Zhou L (2020). OncotRF: an online resource for exploration of tRNA-derived fragments in human cancers. RNA Biol.

[138] Zuo Y, Zhu L, Guo Z (2021). tsRBase: a comprehensive database for expression and function of tsRNAs in multiple species. Nucleic Acids Res.

